# The haemodynamic envelope, a novel aviation-inspired safety framework for personalized monitoring in cardiogenic shock

**DOI:** 10.1093/ehjacc/zuag031

**Published:** 2026-03-09

**Authors:** Christiaan Lucas Meuwese, Joris Anthonius Melkert, Jacob Eifer Møller

**Affiliations:** Department of Cardiology, Thorax Center, Cardiovascular Institute, Erasmus MC, the Netherlands; Department of Intensive Care Adults, Erasmus Medical Center, Rotterdam, The Netherlands; Faculty of Aerospace Engineering, Delft University of Technology, Delft, the Netherlands; Department of Cardiology, Copenhagen University Hospital, Rigshospitalet, Copenhagen, Denmark

**Keywords:** Acute heart failure, Personalized therapy, Flight safety, haemodynamic envelope, Flight envelope

## Abstract

Mortality in severe forms of cardiogenic shock remains above 50%, despite substantial advances in mechanical circulatory support strategies that have enabled clinicians to manage levels of patient complexity once thought unattainable. A key obstacle to effectively implementing and individualizing these sophisticated treatments lies in the continued reliance on outdated safety monitoring strategies that apply uniform cut-off values to a limited set of clinical variables. Such approaches fail to account for the dynamic and patient-specific nature of ‘safe’ haemodynamics. To overcome this, we propose an aviation-inspired safety framework, referred to by ‘the haemodynamic envelope’, designed to continuously and automatically compute patient- and time-specific thresholds across nine interrelated dimensions. This framework defines a dynamic safety window analogous to the systems that transformed flight safety.

## Introduction

Mortality rates in severe forms of cardiogenic shock (CS) have remained in excess of 50%,^1^ prompting experts in the field to advocate for a shift in treatment paradigm.^2^ Advances in mechanical circulatory support (MCS) technology, particularly micro-axial flow pumps,^3^ have expanded treatment options for complex patients. However, effectively implementing these increasingly complicated treatment approaches requires next levels of safety monitoring.

Contemporary patient monitoring systems fall short as they rely on one-size-fits-all cut-offs for a handful of indirect parameters and importantly ignore patient-specific variability, parameter interactions, and temporal changes. For instance, cardiac index cut-off values of 1.8 and 2.2 L/min/m^2^, indicating relative and absolute risk respectively, are applied uniformly to all patients regardless of their systemic oxygen consumption (VO_2_) (*Figure 1*, panel A).

**Figure 1:**
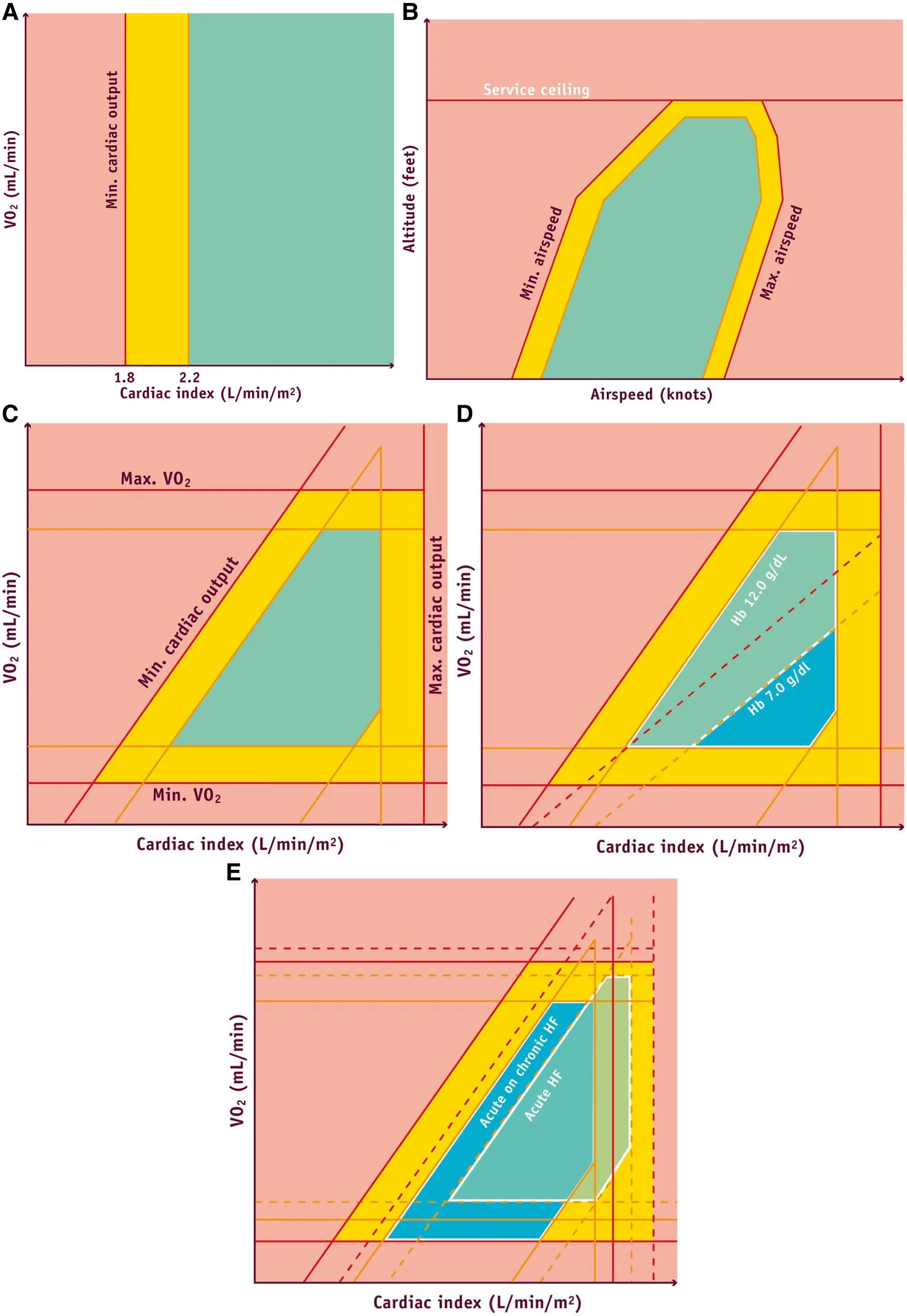
In contemporary clinical practice, uniform cut-off values for cardiac index are applied to all patients regardless of their systemic oxygen consumption (VO_2_) (panel A). The aviation-based *flight envelope* concept (panel B) inspired the development of the *haemodynamic envelope* framework, illustrated in a simplified two-dimensional representation in panel C as a function of VO_2_ and cardiac index. In this model, the influence of varying haemoglobin concentrations is shown in panel D: lower haemoglobin levels (e.g. 7.0 vs. 12.0 g/dL) constrict the safe region within the haemodynamic envelope. Panel E demonstrates how a patient with acute-on-chronic heart failure, having adapted to lower VO_2_ levels compared with an individual experiencing acute heart failure, exhibits a leftward shift and opening of the haemodynamic envelope.

To overcome this critical knowledge gap, we propose a novel aviation-inspired safety framework, denoted as ‘the haemodynamic envelope’ which would allow for the calculation of continuously updated, patient- and disease-specific safety thresholds for nine interrelated dimensions, together defining haemodynamic safety.

## The parallel of CS with aviation

The current landscape in CS shares many similarities with earlier developments in aviation where human limitations in handling increasingly complex aircraft systems were overcome by the development of ‘flight control-’ and ‘autopilot-’ systems. These systems, which serve as the interface between pilots and their aircraft, are essential for operating modern airplanes, and have thereby revolutionized flight safety.^4^

The foundation of these flight control systems is formed by detailed knowledge about an aircraft’s performance limits as a function of altitude, air speed, gravitational loads during ascent or descent, and wind conditions (panel B). These factors dynamically interact to determine both relative- and absolute-thresholds beyond which safe flight is no longer possible. These thresholds are established through theoretical calculations, simulation studies, and ultimately validated by operational flight testing forming a unique ‘flight envelope’ for each aircraft type.^4^ Exceeding flight envelope limits markedly increases the risk of aerodynamic stall, overloading and/or structural failure. For this reason, modern aircraft are equipped with automated ‘envelope protection algorithms’ integrated into their autopilot systems, ensuring that the aircraft remains within its defined flight envelope at all times. Pilots continuously monitor and receive alerts from the onboard safety systems regarding envelope limits throughout the flight, intervening when necessary.

## The haemodynamic envelope framework

The concept of the flight envelope inspired us in the development of the ‘haemodynamic envelope’ framework, which—analogous to an aircraft’s flight envelope—defines individualized physiological limits. We identified the following nine variables (axes) of this framework: (i) systemic oxygen consumption (VO_2_), (ii) cardiac output, (iii) transpulmonary blood flow (being relevant in the setting of venoarterial extracorporeal membrane oxygenation support), (iv) degree of organ/tissue perfusion, (v) mean arterial blood pressure, (vi) left ventricular diastolic filling pressures, (vii) right ventricular diastolic filling pressures, (viii) arterial oxygen saturation, and (ix) haemoglobin concentrations. The selection of these variables was done on theoretical grounds based on the physiological balance between systemic oxygen delivery (DO_2_) and consumption (VO_2_) expressed in Fick’s equation, the principles of Laplace’s law and contemporary clinical possibilities. Additionally, these factors are assumed to capture the significant influences of other relevant variables, including age, comorbidities, metabolic adaptation, and also acute intercurrent events such as an infection or cardiac tamponade. However, the model is designed in a modular manner, allowing for future expansion and integration of additional parameters.

We elected to depict the haemodynamic envelope framework in a simplified, two-dimensional format, as a fully multidimensional representation would introduce unnecessary complexity and detract from our primary objective of presenting a novel conceptual framework. In panel C, the haemodynamic envelope is illustrated as a function of VO_2_ (mL O_2_/min) and cardiac index (L/min/m^2^), representing two out of nine dimensions (axes). The boundaries of the framework are defined by the maximum and minimum VO_2_ values at the upper and lower edges of the diagram, while the left and right limits are determined by the minimum and maximum indexed cardiac output. The blue area denotes a haemodynamically safe area where DO_2_ matches VO_2_. The orange zones indicate areas where a patient becomes at ‘relative risk’, and the red lines and areas indicate regions of ‘absolute risk’ for deterioration. The thresholds of the envelope are unique for each patient depending on the values of the nine haemodynamic envelope axes. For example, low haemoglobin levels (7.0 g/dL) will narrow the haemodynamic envelope safety area, mandating higher cardiac output for a given VO_2_ (panel D). In a patient with acute-on-chronic heart failure, systemic oxygen consumption has often adapted to lower DO_2_ levels prior to developing CS, slightly expanding the safety zone towards the left compared with the safe region of a patient experiencing acute heart failure (panel E). Conversely, contractile reserve may be reduced, resulting in a modest narrowing of the curve on the right. Many other interactions will yield different versions of these graphs.

## Creating the haemodynamic envelope framework and its potential applications

We believe that the haemodynamic envelope can be developed using a cardiovascular computational physiological model (CPM), which relies on a closed-loop electrical analogue of the cardiovascular system as its foundation. Several existing CPMs (e.g. Aplysia^5^ and HARVI^6^) may potentially serve as a basis for a ‘haemodynamic envelope engine’. These CPMs could do so in three sequential steps: (i) to continuously calculate unknown haemodynamic envelope axes values for each patient using their available input data and known physiological relationships. (ii) To calculate time-dependent, individualized axes thresholds by continuously simulating at what values of all of the nine individual variables, DO_2_ would fall short for a required VO_2_. This deficit should also incorporate a buffer, ensuring that interventions can be implemented timely to prevent deterioration beyond critical thresholds. (iii) To predict the effects of different interventions (such as MCS initiation or pharmacological interventions) for a given patient. The haemodynamic envelope framework could also catalyse the approach of using an MCS-induced ‘haemodynamic buffer’ to reduce inotrope dosages, and facilitate the initiation of heart failure guideline-directed medical therapies (GDMT).^7^ By design, all this information would be individualized and continuously updated over time. The last two steps are based on iterative simulations where the CPM repeatedly simulates what would happen under different scenarios (alternating haemodynamic envelope thresholds or treatment interventions).

It is important to emphasize that this framework is currently theoretical, and empirical data supporting its application are presently lacking. It is the intention of the authors to, over time, develop this approach into actionable methods. Studies should validate CPM based predictions against golden-standard measurements/observations across different degrees of CS severity and underlying aetiologies. Such studies should also identify the optimum safety margin with respect to identified thresholds. CPM performance could potentially be optimized through deep reinforcement learning techniques, yielding more accurate predictions of parameter values and envelope limits. These techniques have also been successful in aviation, such as aircraft landing algorithms and engine monitoring systems.^8^

With ongoing technological advances enabling the measurement of perfusion in individual organs, we anticipate the conception of ‘organ-specific envelopes’, which together would inform the overarching haemodynamic envelope. This concept parallels that of an aircraft, where components such as the landing gear, wings, and fuselage each have distinct envelopes; collectively, these define the boundaries of the aircraft’s overall flight envelope, which varies according to the phase of flight. Looking further ahead, we also envision the development of ‘haemodynamic envelope protection’ algorithms—analogous to those used in modern aviation—that have revolutionized flight safety. For example, such algorithms could automatically adjust MCS blood flow, and vasoactive medication to maintain patients within their individualized haemodynamic envelope boundaries, and ‘learn on the spot’.

## Conclusion

We have introduced a novel, aviation-inspired safety framework—the haemodynamic envelope—which has the potential to bridge the gap between escalating treatment risks and the growing complexity of both patients and medical technologies. Moreover, this framework can unlock a new line of research focused on cardioprotective therapies in CS and related conditions.

## Data Availability

No new data were generated or analysed in support of this research.
